# Identification and Validation of Aging-Related Genes in Idiopathic Pulmonary Fibrosis

**DOI:** 10.3389/fgene.2022.780010

**Published:** 2022-02-08

**Authors:** Jie He, Xiaoyan Li

**Affiliations:** ^1^ Clinical Medical College of Chengdu Medical College, Chengdu, China; ^2^ Department of Respiratory and Critical Care Medicine, The First Affiliated Hospital of Chengdu Medical College, Chengdu, China; ^3^ Department of Endocrinology, The First Affiliated Hospital of Chengdu Medical College, Chengdu, China

**Keywords:** aging, IPF, bioinformatics analysis, gene expression omnibus dataset, gene

## Abstract

Aging plays a significant role in the occurrence and development of idiopathic pulmonary fibrosis (IPF). In this study, we aimed to identify and verify potential aging-associated genes involved in IPF using bioinformatic analysis. The mRNA expression profile dataset GSE150910 available in the Gene Expression Omnibus (GEO) database and R software were used to identify the differentially expressed aging-related genes involved in IPF. Hub gene expression was validated by other GEO datasets. Gene ontology (GO) enrichment analysis and Kyoto Encyclopedia of Genes and Genomes (KEGG) pathway enrichment analysis were performed on differentially expressed aging-related genes. Subsequently, aging-related genes were further screened using three techniques (least absolute shrinkage and selection operator (LASSO) regression, support vector machine, and random forest), and the receiver operating characteristic curves were plotted based on screening results. Finally, real-time quantitative polymerase chain reaction (qRT-PCR) was performed to verify the RNA expression of the six differentially expressed aging-related genes using the blood samples of patients with IPF and healthy individuals. Sixteen differentially expressed aging-related genes were detected, of which the expression of 12 were upregulated and four were downregulated. GO and KEGG enrichment analyses indicated the presence of several enriched terms related to senescence and apoptotic mitochondrial changes. Further screening by LASSO regression, support vector machine, and random forest identified six genes (*IGF1, RET, IGFBP2, CDKN2A, JUN,* and *TFAP2A*) that could serve as potential diagnostic biomarkers for IPF. Furthermore, qRT-PCR analysis indicated that among the above-mentioned six aging-related genes, only the expression levels of *IGF1, RET,* and *IGFBP2* in patients with IPF and healthy individuals were consistent with the results of bioinformatic analysis. In conclusion, bioinformatics analysis identified 16 potential aging-related genes associated with IPF, and clinical sample validation suggested that among these, *IGF1, RET,* and *IGFBP2* might play a role in the incidence and prognosis of IPF. Our findings may help understand the pathogenesis of IPF.

## Introduction

Idiopathic pulmonary fibrosis (IPF) is a type of chronic, progressive, fibrosing interstitial pneumonia, and its etiology and pathogenesis are not yet fully understood (Richeldi et al., 2017). Its key pathological characteristics include alveolar epithelial cell (AEC) injury, inflammatory cell infiltration, massive extracellular matrix accumulation, epithelial–mesenchymal transition, and fibroblast transformation to myofibroblasts. These detrimental pathological effects eventually result in an irreversible and progressive respiratory insufficiency (Reddy et al., 2016; Colunga Biancatelli et al., 2020). Some of the risk factors related to the incidence of IPF include smoking and old age (Hill et al., 2019; Rahaghi et al., 2020), and more men are affected by IPF than women. The occurrence rate of IPF and its related mortality rate increase substantially with age (Ebner et al., 2020; Phan et al., 2021); two-thirds of patients with IPF are aged 60 years or more at the onset of the disease and the average age at the time of diagnosis is 66 years; the estimated prevalence of IPF among individuals over 65 years of age may be as high as 94 per 100,000 people (Hecker et al., 2014; Ryerson et al., 2016; López-Ramírez et al., 2018; Kishaba, 2019; Lee et al., 2020). At present, the key treatment approaches for IPF are based mainly on the symptoms and have low therapeutic effects; the 5 year survival rate after diagnosis is less than 50%, and the median survival time is only 2–3 years (Kistler et al., 2014; Yoshihara et al., 2020). Increasing evidence shows that different biological functions such as cell proliferation, apoptosis, senescence, and autophagy play a role in IPF pathogenesis (Predescu et al., 2017; Cong et al., 2020; Krempaska et al., 2020). Among these, cell senescence plays a major role.

Cell senescence is defined as an irreversible inhibition of cell proliferation wherein the cell cycle is usually arrested permanently in the G0 or G1 phase. Changes in cell morphology include cell flattening, nuclear enlargement, and chromatin aggregation (Anwar et al., 2016). Another prominent feature of senescent cells is that they secrete a variety of cytokines, chemokines, growth factors, and matrix metalloproteinases, thereby constituting the senescence-associated secretory phenotype (SASP) (Huda et al., 2019). Increasing evidence supports a correlation between cellular senescence and IPF pathogenesis. For example, Hecker et al. (2014) showed that persistent fibrosis in the lung tissues of aged mice was characterized by an accumulation of senescent and apoptosis-resistant myofibroblasts, and these mice demonstrated an impaired capacity for fibrosis resolution. Yanai et al. (2015) suggested that cellular senescence could serve as a bridge connecting lung aging and pulmonary fibrosis, and is a crucial factor in disease progression. Álvarez et al. (2017) found that IPF human lung fibroblasts developed senescence leading to decreased apoptosis, and the development of the SASP might be a critical contributor to the fibrotic process observed in IPF. Additionally, some studies have highlighted that a variety of aging-related pathways are activated in epithelial cells, fibroblasts, and progenitor cells in the lungs of patients with IPF (Mora et al., 2017; Chen et al., 2019); these activated pathways promote abnormal secretory phenotype in lung epithelial cells, augment the resistance of myofibroblasts to apoptosis, and accelerate IPF progression. In the lung biopsies of patients with IPF, the level of SA-β-gal, a specific cellular senescence marker, was increased compared to that in patients with chronic obstructive pulmonary disease or hypersensitivity pneumonitis (Kellogg et al., 2021). Kim et al. (2021) evaluated the genomic profile of fibrotic and normal lung tissues and found that the core molecular network of IPF featured p53 signaling pathway and cellular senescence. Nevertheless, it remains unknown which aging-related genes are critical for the development of IPF and thus, the correlation between IPF and aging-related genes is yet to be understood. Further studies are needed to determine new biomarkers for the treatment of IPF based on potential aging-related genes involved in IPF.

GSE150910 is an IPF-related data set with a large sample size (103 IPF lung and 103 normal lung tissues). Furusawa et al. (2020) completed a sequencing analysis of GSE150910 and revealed 1,183 differentially expressed mRNAs between IPF and normal lung tissues. In this study, we aimed to analyze the GSE150910 data set from different perspectives. The differential expression of aging-related IPF genes was determined by bioinformatic methods using limma test, protein-protein interaction (PPI) analysis, correlation analysis, gene ontology (GO) enrichment analysis, and Kyoto Encyclopedia of Genes and Genomes (KEGG) pathway enrichment analysis. Then, machine learning approaches were used for extensive filtration and diagnostic IPF molecular marker identification. Finally, the expression levels of aging-related genes screened by machine learning approaches was validated using other Gene Expression Omnibus (GEO) datasets and clinical samples.

## Materials and Methods

### Aging-Related Gene Data Set and Sequencing Data

For this study, 307 genes were selected from Human Aging Genomic Resources (https://genomics.senescence.info/) (Supplementary Table S1). The GSE150910 (Furusawa et al., 2020) mRNA expression profile dataset was downloaded from GEO (https://www.ncbi.nlm.nih.gov/geo/). The GSE150910 dataset, which contains 103 IPF specimens and 103 normal lung tissue specimens, is based on GPL24676 platform (Illumina Nova Seq 6000, *Homo sapiens*).

### Differential Expression Analysis of Aging-Related Genes

For the RNA sequencing analysis of the GSE150910 data set, gene expression levels were normalized using Transcripts Per kilobase Million (TPM) values; the following formula was used: TPM = Read count × 1,000,000/Mapped Reads (Jiao et al., 2018). Principal component analysis (PCA) verified the repeatability of the GSE150910 data. Perl software, version 5.20.2 (Perl Foundation, Holland, MI, United States) was used to retrieve the aging-related gene expression matrix from the GSE150910 dataset. The “limma” software package helped identify differentially expressed aging-related genes. The Wilcoxon rank sum test was used to analyze the significance of differential aging-related gene expression, with an adjusted value of *p* < 0.05 and an absolute value of log2 (fold change [FC]) > 1. The exact formulae and codes are provided in Supplementary File S1. The “heatmap” and “ggplot2” software packages of R software were used to draw heat maps, volcano maps, and box plots.

### Protein-Protein Interactions and Correlation Analyses of Differentially Expressed Aging-Related Genes

STRING database (https://string-db.org/) and Cytoscape software (version 3.8.1) were used to observe the interactions between the differentially expressed aging-related genes. Pearson correlation analysis function in the R software “corrplot” package was used to identify the correlation between the differentially expressed aging-related genes.

### Gene Ontology and Kyoto Encyclopedia of Genes and Genomes Enrichment Analyses of Aging-Related Genes

The GO and KEGG pathway enrichment analysis was performed using the “GO plot” software package in the R software. GO analysis included cell composition, biological process, and molecular function.

### Screening Aging-Related Genes Through Least Absolute Shrinkage and Selection Operator Logistic Regression, Support Vector Machine Recursive Feature Elimination, and Random Forest

Software package “glmnet” (McEligot et al., 2020) was used to perform LASSO logistic regression analysis on the identified aging-related genes, and the small sample size and the large number of variables acquired were considered. LASSO is a statistical technique with the dual features of subset selection and ridge regression. It implements ordinary least squares, but the sum of the absolute values of the regression coefficients is less than the predetermined constant value (Pierre et al., 2020). Logistic regression LASSO is a generalization of the output variable LASSO with a binomial distribution. Using LASSO, some regression coefficients are reduced to zero, so only variable genes with non-zero regression coefficients remain as a part of the model. Here, the acquired aging-related genes were further narrowed down using LASSO. Furthermore, a machine learning technique, known as SVM-RFE, which works on the principle of support vector machines, was utilized to find the best variable by deleting the feature vector generated by SVM (Sundermann et al., 2017). The SVM module was set up using the “e1071” software package to further screen aging-related genes in IPF. Finally, the genes at the intersection of those screened by LASSO and SVM-RFE were used for the diagnostic analysis of IPF, and a receiver operating characteristic (ROC) curve was drawn. The obtained genes were considered aging-related hub genes. Random forest is an algorithm based on the construction of a binary tree using recursive partitioning (Macedo Hair et al., 2019). The number of trees in the random forest algorithm was set to 500, and the Gini index was used as an importance measure (Yan et al., 2020). Thus, we used the random algorithm to sort the aging-related hub genes by the mean decrease in Gini index. Random forest classification models were built using the “randomForest” package in R software with genes (features) in columns and samples in rows.

### Validation of Aging-Related Hub Genes in Other Datasets

Expression patterns of hub aging-related genes were validated in seven independent datasets [GSE10667 (Konishi et al., 2009), GSE24206 (Meltzer et al., 2011), GSE73189 (Yu et al., 2017), GSE28042 (Huang et al., 2015), GSE32537 (Yang et al., 2013), GSE21369 (Cho et al., 2011), and GSE110147 (Cecchini et al., 2018)] by comparing the data of healthy controls and patients with IPF. Detailed information on these datasets is presented in Supplementary Table S2. The microarray data of GSE32537, based on GPL6244, included 119 lung tissues with IPF and was used to validate the diagnostic efficacy of aging-related hub genes.

### Patients With Idiopathic Pulmonary Fibrosis and Healthy Individuals

Twenty patients with IPF (case group) and age-matched healthy individuals (control group) were enrolled at the First Affiliated Hospital of Chengdu Medical College from July 2018 to July 2021. The enrollment criteria for patients in this study were similar to the published IPF criteria (Lynch et al., 2018; Raghu et al., 2018). The selection criteria for IPF included patients who showed a possible common pattern of interstitial pneumonia or had prominent features of interstitial pneumonia on high-resolution computed tomography images. Patients with other known causes of interstitial lung disease (such as connective tissue disease with autoimmune characteristics, family or occupational environmental exposure, and drug toxicity) were excluded.

Twenty healthy individuals in control group were recruited at the health examination center of the hospital. This study was approved by the Ethics Committee of the First Affiliated Hospital of Chengdu Medical College (Ethics number, 2021CYFYIRB-BA-32-01) and was conducted according to the tenets of the Declaration of Helsinki (World Medical Association, 2013). All participants provided informed consent for participation.

### RNA Extraction and Real-Time Quantitative Polymerase Chain Reaction

Peripheral blood mononuclear cells (PBMCs) were obtained from the blood samples of patients using Ficoll solution (Solarbio Life Sciences, Beijing, China). Total RNA was extracted from the isolated PBMCs using an RNA extraction kit (Omega, Guangzhou, China). The mRNA levels were detected using the TB Green PreMix Ex Taq Kit (Takara, Dalian, China), and reverse transcription was performed using PrimeScript RT Master Mix Kit (Takara, Dalian, China). The primer sequences are listed in Supplementary Table S3. The 2^-△△Ct^ method was used to assess relative mRNA expression normalized to GAPDH mRNA levels.

### Statistical Analysis

Statistical analyses were performed using R software (version 3.6.1, http://www.R-project.org) and GraphPad Prism version 8 (GraphPad Software, La Jolla, CA). The Wilcoxon rank sum test was used to analyze the significance of the differential aging-related gene expression in the GEO datasets. Student’s *t*-test was performed to compare gene expression levels of clinical specimens. Statistical significance was set at *p* < 0.05. MedCalc software (MedCalc Software Ltd., Ostend, Belgium) was used to analyze the data and draw the ROC curve.

## Results

### Differentially Expressed Aging-Related Genes Based on IPF-Retrospective Analysis

**FIGURE 1:**
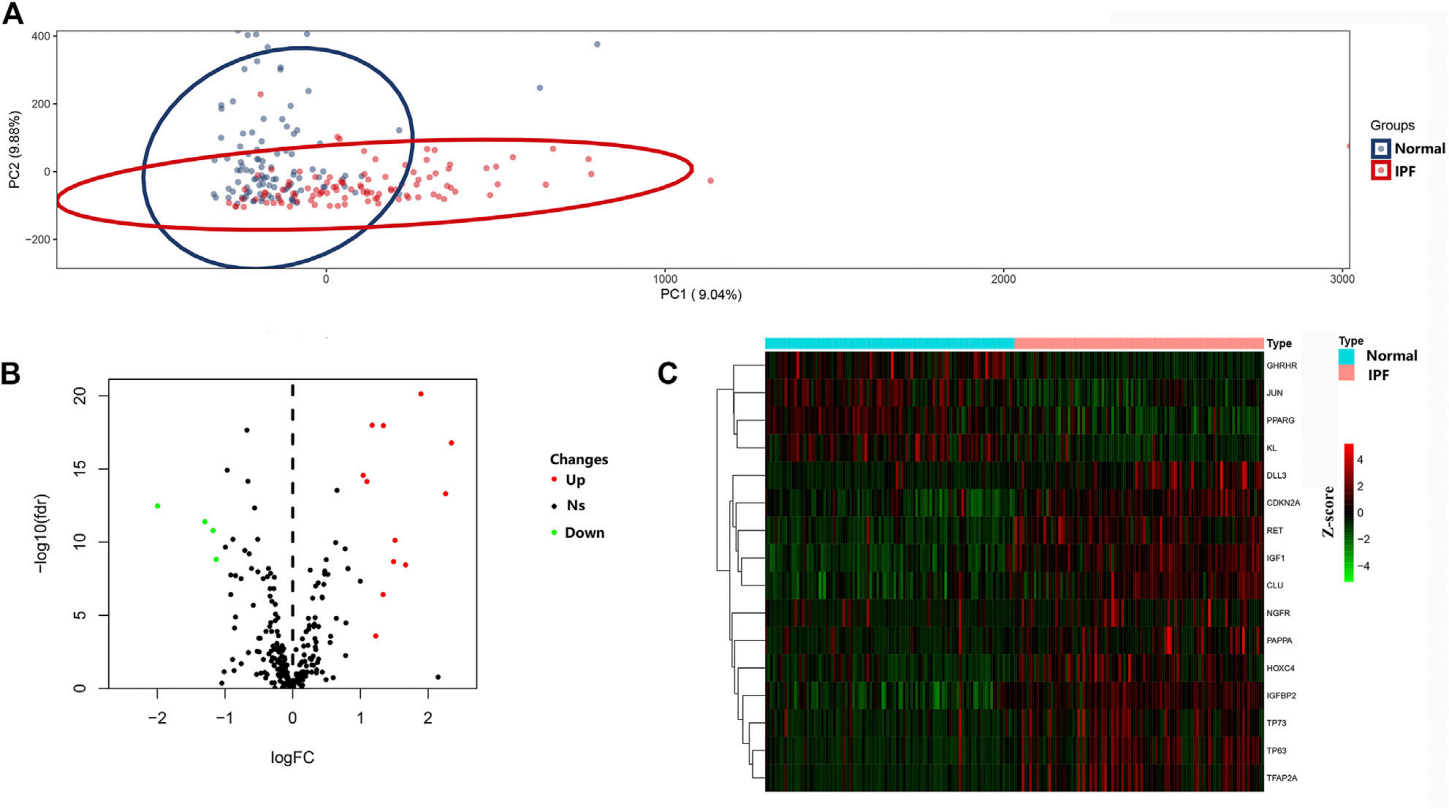
Differential expressed aging-related genes in IPF and healthy samples. **(A)** Principal component analysis for GSE150910. **(B)** Volcano of the 307 differentially expressed aging-related genes. The red dots represent the significantly up-regulated genes and the blue suggest the significantly down-regulated genes. **(C)** Heatmap of the 16 differentially expressed aging-related genes in IPF and healthy samples.

**FIGURE 2:**
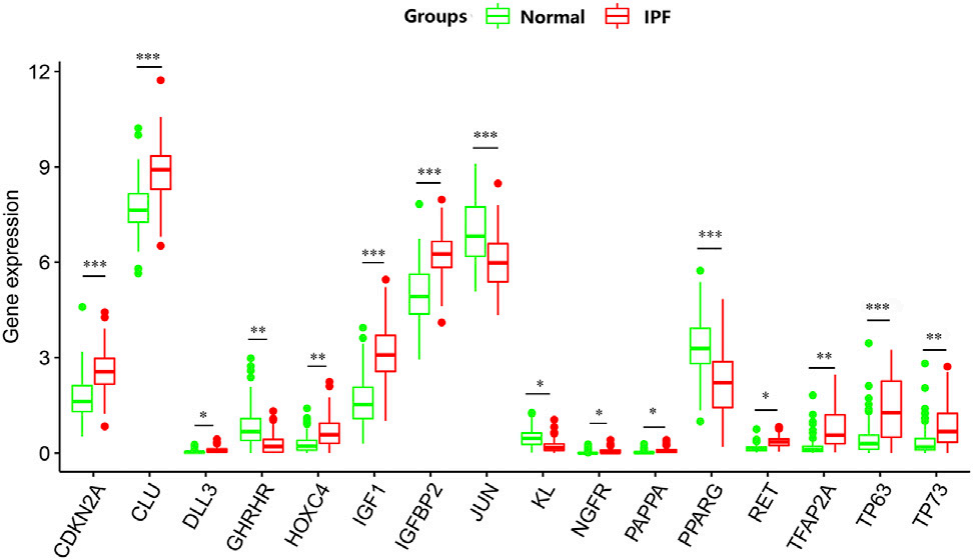
The box plot of 16 differentially expressed aging-related genes in IPF and healthy samples.*p < 0.05; **p < 0.01; ***p < 0.005.

PCA was conducted to evaluate the repeatability of data within the group and showed that GSE150910 had a good data repeatability (Figure 1A). Notably, PCA also revealed that one of the IPF samples was an outlier, and accordingly, this outlier was excluded from analysis. Subsequently, differential gene analysis was performed using 307 aging-related genes in 102 cases of IPF lung tissues and 103 cases of normal lung tissues using an adjusted *p* value of <0.05 and an FC absolute value of >1 as the standard. A total of 16 aging-related genes, including 12 genes with upregulated expression and four genes with downregulated expression, were identified (Table 1). These 16 aging-related genes differentially expressed between IPF group and control group are displayed in the heat map and volcano map (Figures 1B,C). The box plot highlights their pattern of expression in the IPF samples and normal controls (Figure 2). *TFAP2A, TP63,* and *IGF1* were the top three genes with upregulated expression, while *GHRHR, KL,* and *PPARG* were the top three genes with downregulated expression.

**TABLE 1:** The 16 differentially expressed aging-related genes in IPF samples compared to healthy samples.

Gene symbol	Log_2_FC	Changes	*p*-value	Adjusted. *p*-value	Chromosome
GHRHR	−1.997,412,344	Down	1.67E-14	3.36E-13	7p14.3
KL	−1.298,550,570	Down	2.29E-13	3.96E-12	13q13.1
PPARG	−1.177,922,209	Down	1.01E-12	1.62E-11	3p25.2
JUN	−1.132,334,945	Down	1.48E-10	1.49E-09	1p32.1
CDKN2A	1.040,858,139	Up	8.00E-17	2.77E-15	9p21.3
CLU	1.096,936,165	Up	2.64E-16	7.11E-15	8p21.1
IGFBP2	1.172,220,971	Up	1.27E-20	1.03E-18	2q35
NGFR	1.226,321,745	Up	8.42E-05	0.00025	17q21.33
PAPPA	1.333,661,194	Up	7.84E-08	3.72E-07	9q33.1
RET	1.338,350,201	Up	1.77E-20	1.07E-18	10q11.21
TP73	1.488,719,589	Up	2.22E-10	2.06E-09	1p36.32
HOXC4	1.510,110,746	Up	5.61E-12	7.54E-11	12q13.13
DLL3	1.667,316,642	Up	3.92E-10	3.52E-09	19q13.2
IGF1	1.893,881,384	Up	3.01E-23	7.28E-21	12q23.2
TP63	2.256,195,667	Up	2.23E-15	4.90E-14	3q28
TFAP2A	2.516,340,767	Up	2.21E-19	1.07E-17	6p24.3

### Protein-Protein Interactions Network and Correlation Analyses of Differentially Expressed Aging-Related Genes

**FIGURE 3:**
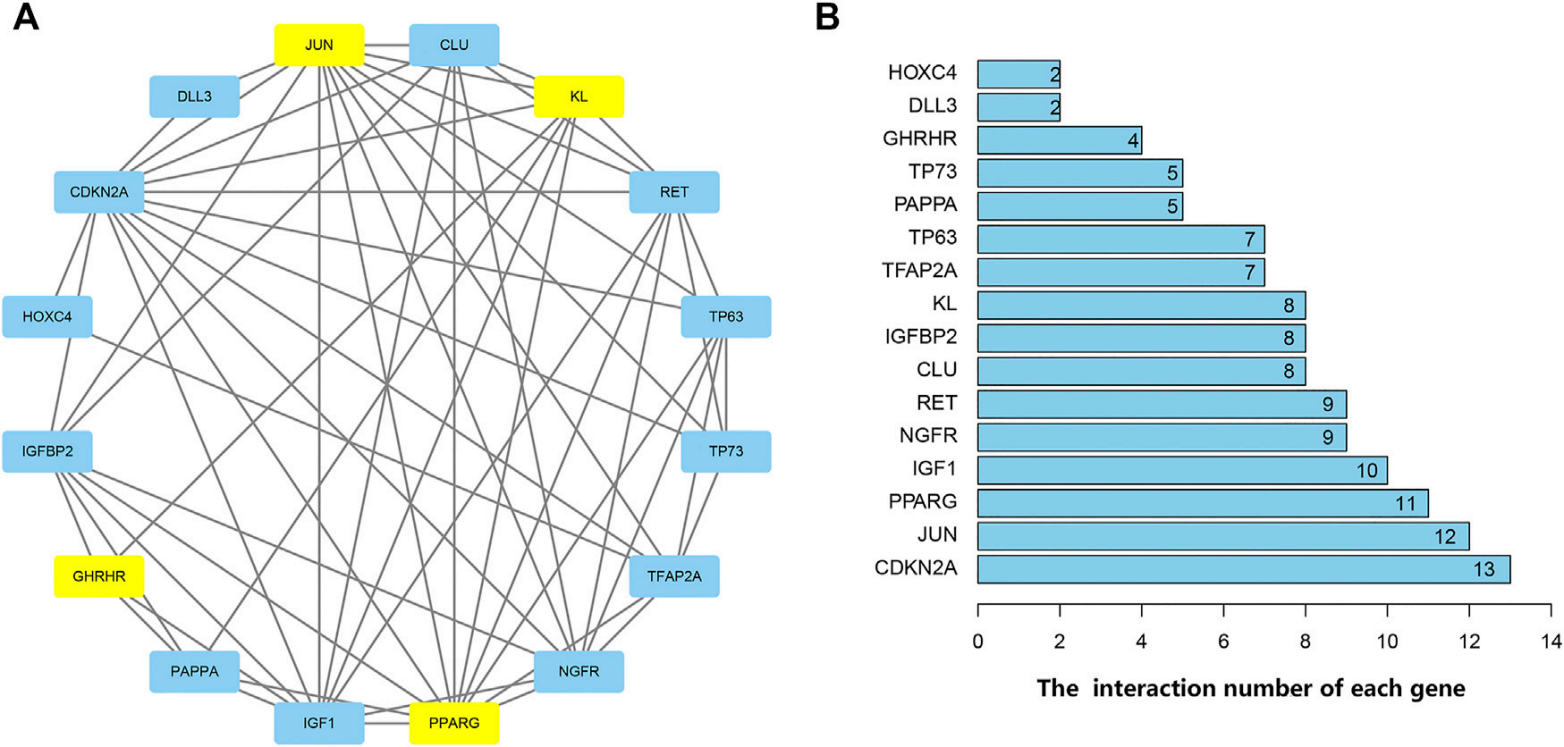
Protein-protein interactions (PPI) analysis the 16 differentially expressed aging-related genes. **(A)** The PPI among 16 differentially expressed aging-related genes. The blue represents the significantly up-regulated genes and the yellow suggests the significantly down-regulated genes. **(B)** The interaction number of each differentially expressed aging-related gene.

**FIGURE 4:**
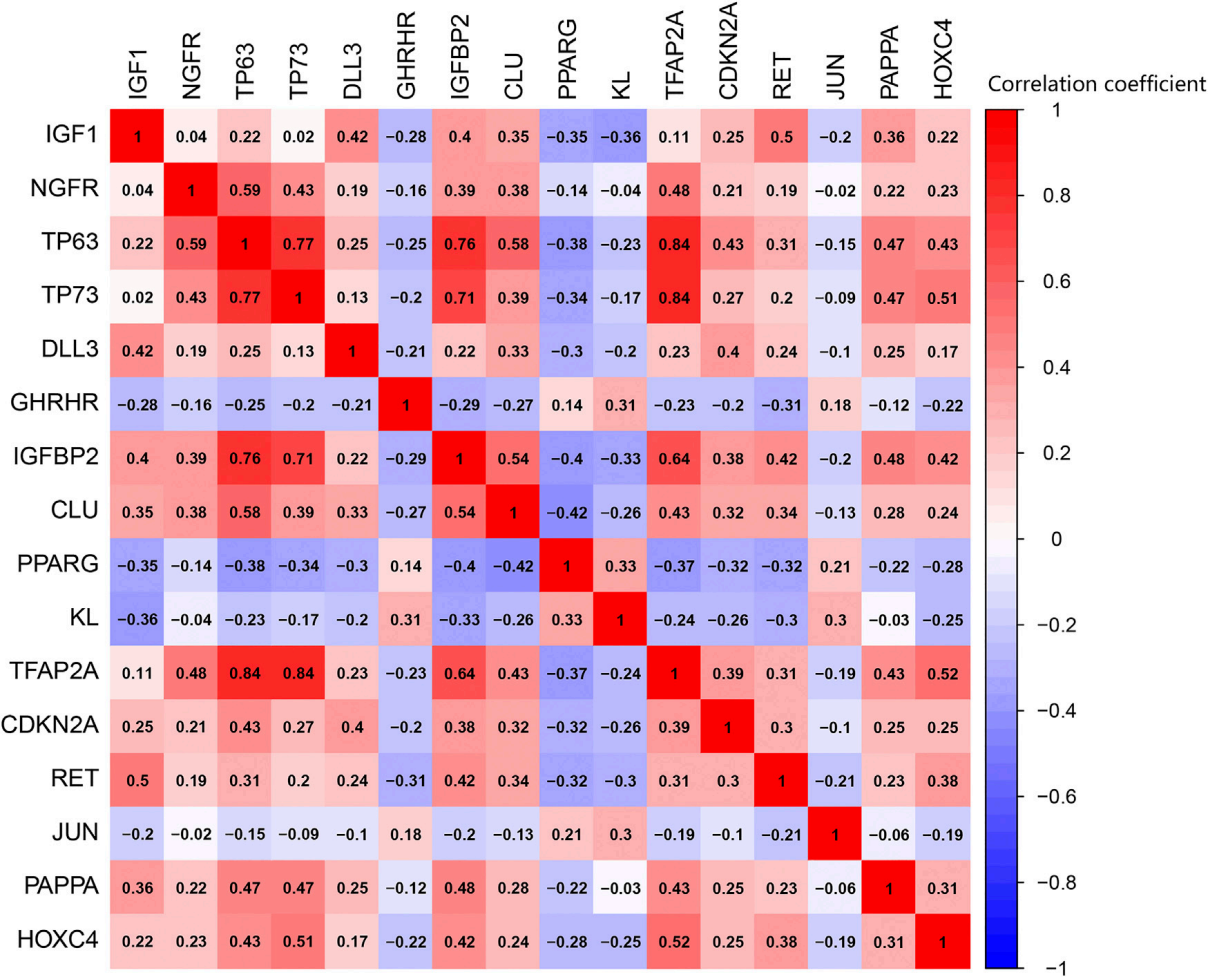
Pearson correlation analysis of the 16 differentially expressed aging-related genes.

PPI analysis revealed the interactions between these aging-related genes (Figure 3A) and identified the number of interactions for each gene (Figure 3B). Correlation analysis indicated a correlation between 16 differentially expressed aging-related genes in the GSE150910 dataset (Figure 4).

### Gene Ontology and Kyoto Encyclopedia of Genes and Genomes Enrichment Analyses of Differentially Expressed Aging-Related Genes

**FIGURE 5:**
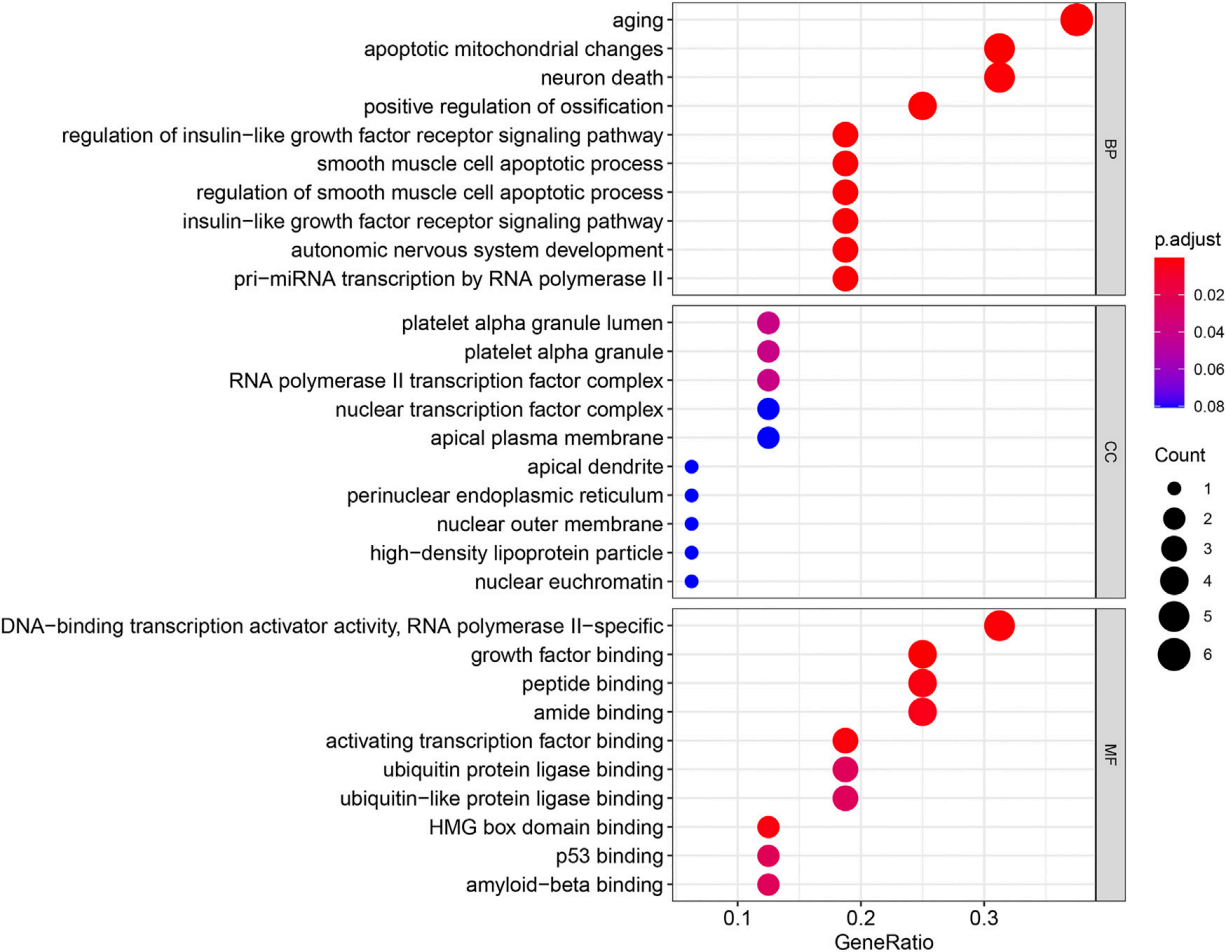
Gene Ontology (GO) enrichment analysis of 16 differentially expressed aging-related genes. Abbreviations: BP, biological process; CC, cellular component; MF, molecular function.

**FIGURE 6:**
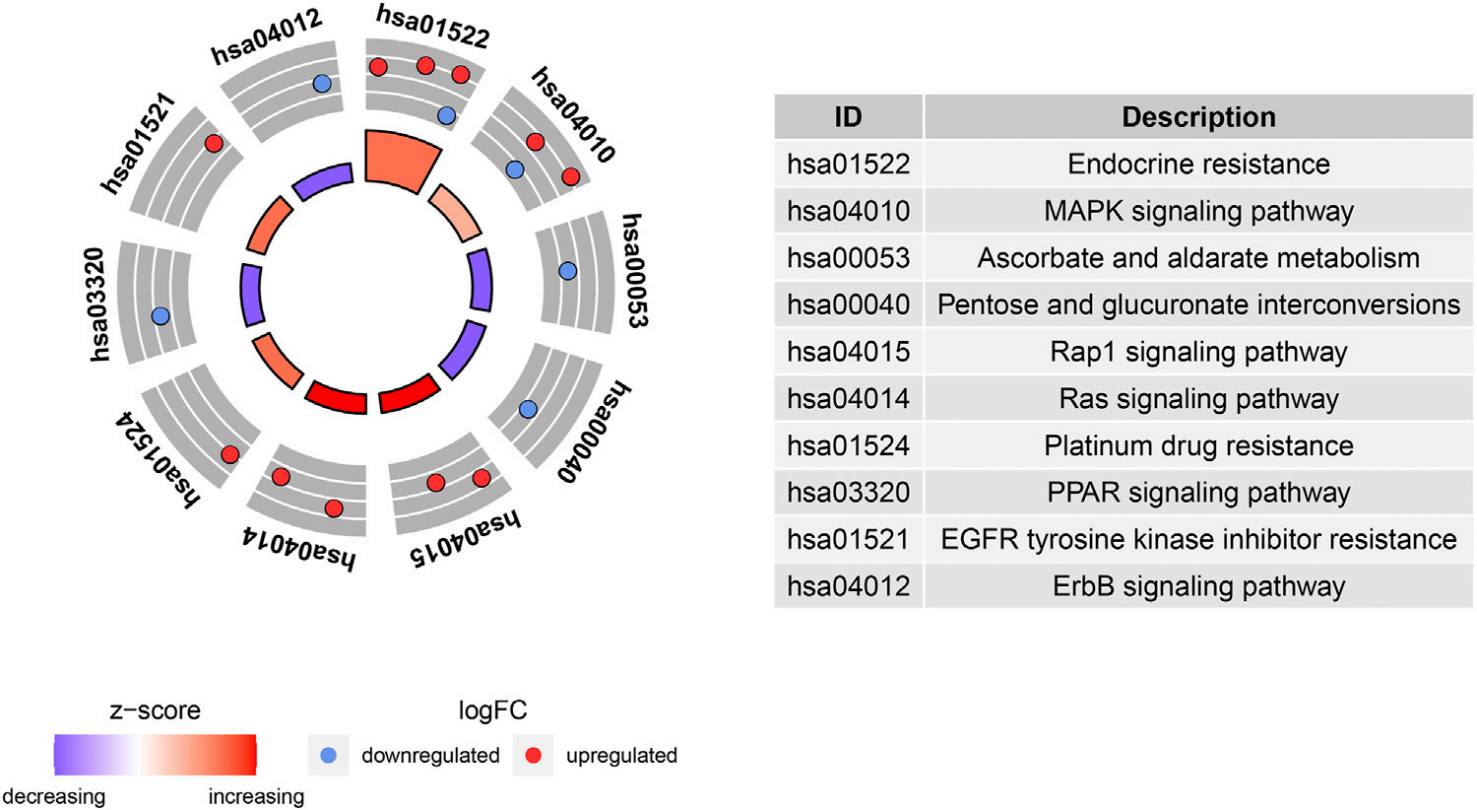
Kyoto Encyclopedia of Genes and Genomes (KEGG) analysis of 16 differentially expressed aging-related genes.

GO and KEGG enrichment analyses were performed using the R software to determine the potential biological functions of differentially expressed aging-related genes. The most significant enrichment terms for GO were aging, apoptosis, mitochondrial changes, neuronal death (biological process), platelet alpha granule lumen, platelet alpha granule, RNA polymerase II transcription factor complex (cellular component), DNA-binding transcription activator activity, RNA polymerase II−specific, growth factor binding, and peptide binding (molecular function) (Figure 5). The KEGG enrichment analysis showed that the differentially expressed aging-related genes played a key role in endocrine resistance and the MAPK signaling pathway (Figure 6).

### Genes Screened by Least Absolute Shrinkage and Selection Operator Logistic Regression, Support Vector Machine Recursive Feature Elimination , and Random Forest

**FIGURE 7:**
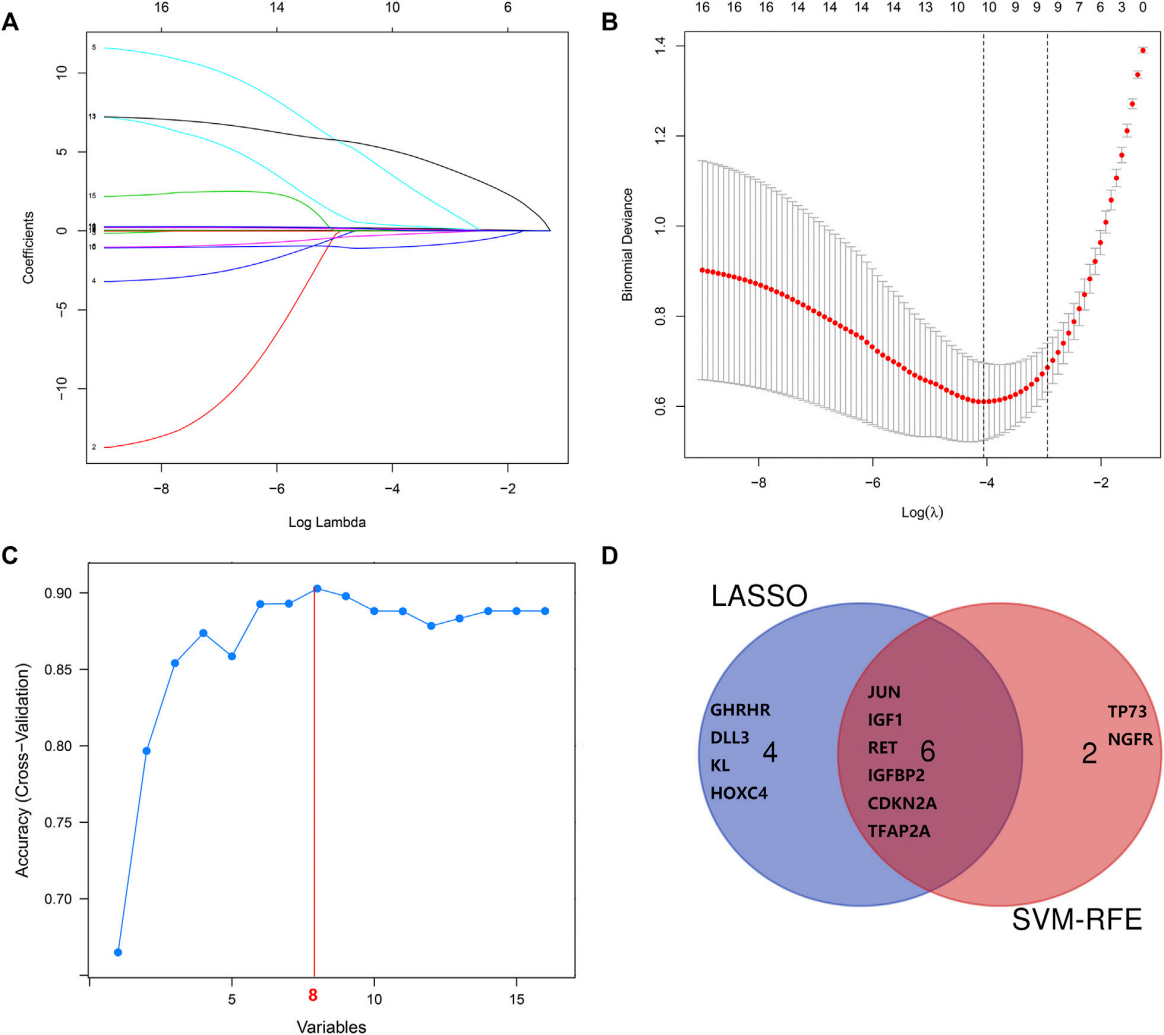
Screening and verification of diagnostic markers. **(A,B)** Least absolute shrinkage and selection operator (LASSO) logistic regression algorithm to screen diagnostic markers. **(C)** Support vector machine-recursive feature elimination (SVM-RFE) algorithm to screen diagnostic markers. **(D)** Venn diagram demonstrates the intersection of diagnostic markers obtained by the two algorithms.

The LASSO regression algorithm identified 10 out of the 16 aging-related genes (the optimal sparseness parameter λ was 0.017) (Figures 7A,B), whereas the SVM-RFE algorithm identified eight genes out of the 16 aging-related genes (Figure 7C). The six genes commonly identified in the results of the two algorithms comprised *IGF1*, *CDKN2A*, *JUN*, *IGFBP2*, *RET*, and *TFAP2A* (Figure 7D). We applied the random forest algorithm to construct 500 decision trees, from which a relatively stable out-of-bag classification error rate of 11.65% was obtained (Supplementary Figure S1A). The random forest analysis showed that these six aging-related hub genes were also the top-ranked genes (Supplementary Figure S1B).

### Validation of Aging-Related Hub Genes in Other Datasets

We validated the gene expression patterns in seven independent datasets (GSE10667, GSE24206, GSE73189, GSE28042, GSE32537, GSE21369, and GSE110147) using the “limma” test. As shown in Supplementary Figure S2, overexpression of *IGF1*, *CDKN2A*, *IGFBP2*, *RET*, and *TFAP2A* and underexpression of *JUN* was detected in IPF samples. This gene expression trend was consistent with that observed in GSE150910.

### Receiver Operating Characteristic Curves of Six Specifically Expressed Aging-Related Genes in the Idiopathic Pulmonary Fibrosis and Normal Lung Tissues

**FIGURE 8:**
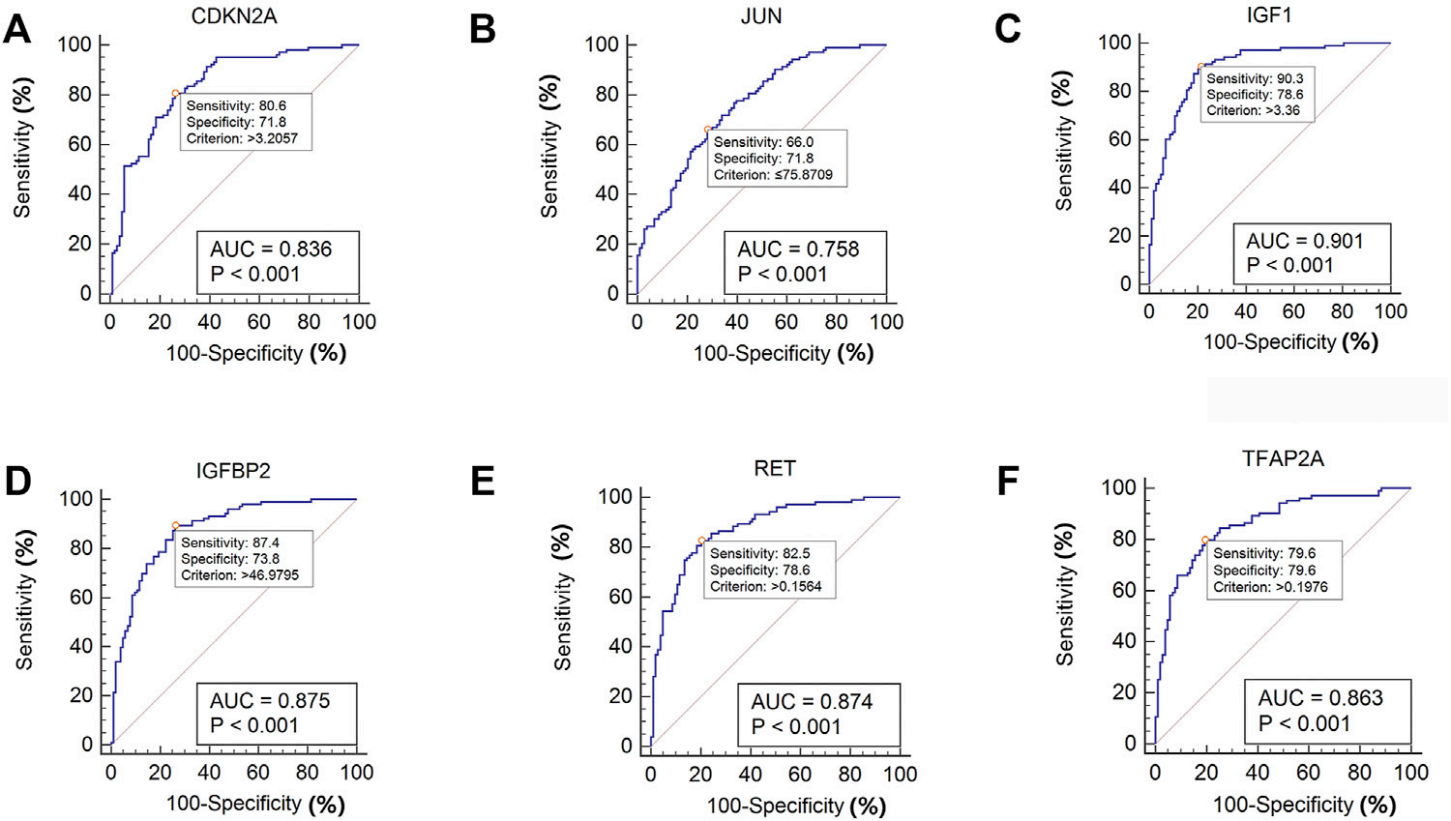
ROC curve of the six specifically expressed hub genes in IPF and healthy samples.

MedCalc software was used to analyze the expression of the six aging-related genes in the IPF and normal lung samples of the GSE150910 dataset, and ROC curves were drawn. The area under the curve (AUC) combines sensitivity and specificity, and can authenticate the inherent validity of a diagnostic test (Kumar and Indrayan, 2011). The six specifically expressed aging-related genes had a higher diagnostic value for IPF. Of these, *IGF1* showed the highest diagnostic value in IPF samples (AUC = 0.901). The diagnostic values of the other genes were as follows: *CDKN2A* (AUC = 0.826), *JUN* (AUC = 0.758), *IGFBP2* (AUC = 0.875), *RET* (AUC = 0.874), and *TFAP2A* (AUC = 0.8863) (Figure 8). These six genes could be considered as potential diagnostic biomarkers for IPF. The diagnostic efficacy of these six hub aging-related genes was validated using the GSE32537 dataset (Supplementary Figure S3).

### Verification of the Differentially Expressed Aging-Related Genes in Clinical Samples

**FIGURE 9:**
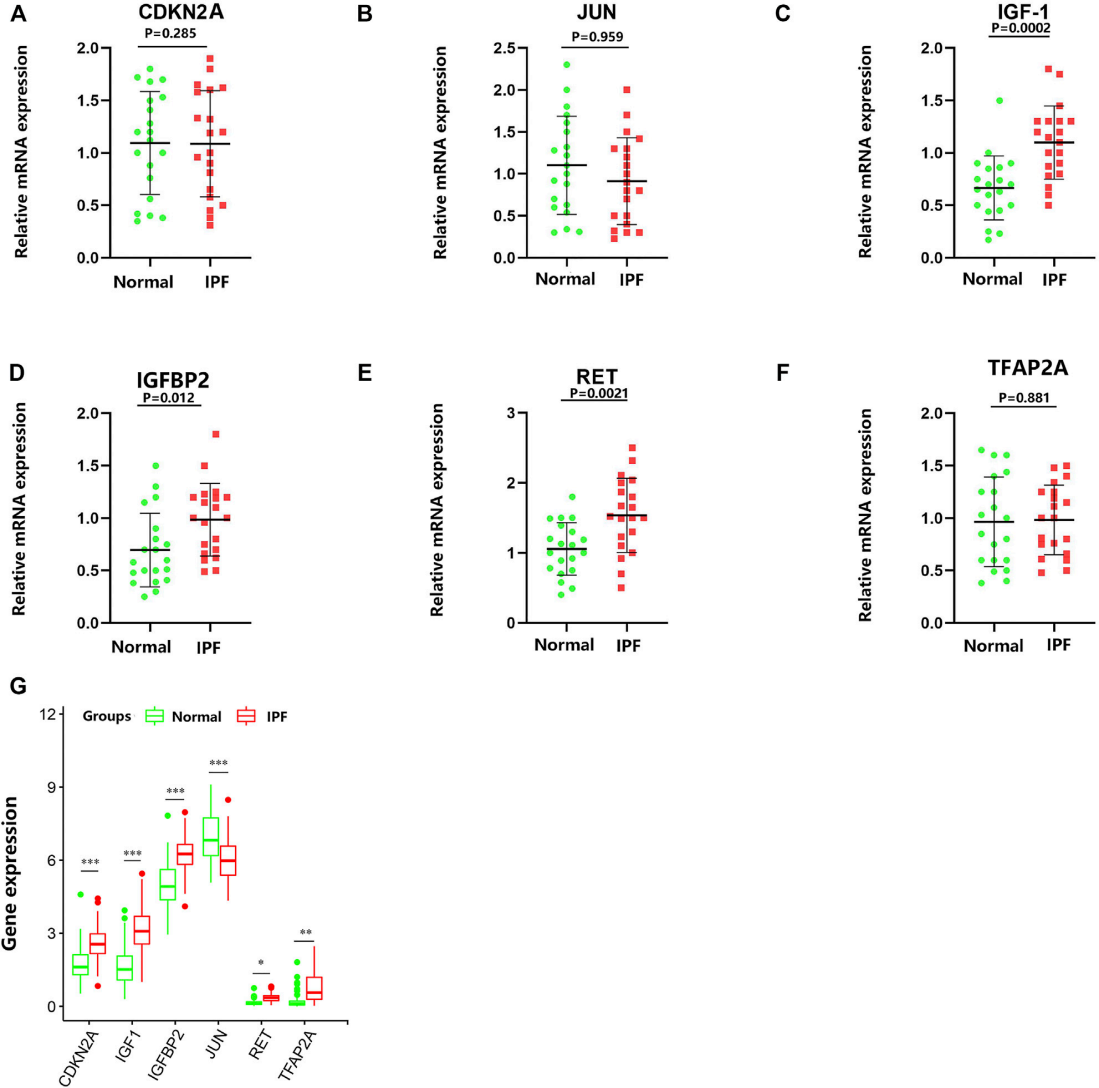
RNA expression of six specifically expressed aging-related genes were measured in IPF and healthy samples. *p*-values were calculated using a two-sided unpaired Student’s t-test. **(A–F)** Relative mRNA expression of the aging-related genes was analyzed by qRT-PCR. **(G)** The RNA-Seq expression of aging-related genes. ^*^ *p* < 0.05; ^**^ *p* < 0.01; ^***^ *p* < 0.005.

To verify the reliability of the GSE150910 dataset, the expression of the above-mentioned six aging-related genes was further analyzed by performing qRT-PCR using the clinical samples. Table 2 summarizes the clinicopathological variables of the case and control groups. Our clinical validation trial demonstrated that the results of the analysis were broadly similar to those of the main bioinformatic analysis. The expression levels of *IGF1* (*p* = 0.0002), *RET* (*p* = 0.0021), and *IGFBP2* (*p* = 0.012) were significantly higher in IPF blood samples than in normal blood samples, while those of *CDKN2A*, *JUN*, and *TFAP2A* (*p* values are all greater than 0.05) were comparable between the two groups (Figure 9).

**TABLE 2:** Clinicopathological variables of IPF and Controls in this study.

Variables	IPF (20)	Control (20)	*p*-value
Age (years)	60.34 ± 5.82	62.11 ± 4.04	0.271
Gender (male/female)	11/9	10/10	0.751
BMI (kg/m2)	26.83 ± 5.51	24.33 ± 4.78	0.133
Smokers (NS/FS/S)	5/5/10	4/7/9	0.779
FEV1% pred	113.45 ± 12.58	70.03 ± 15.33	<0.001
FVC% pred	115.34 ± 14.86	68.61 ± 16.05	<0.001
FEV1/FVC%	80.96 ± 4.87	75.06 ± 12.39	0.052
DLCO% pred	nd	37.82 ± 15.34	—
KCO% pred	nd	57.65 ± 16.77	—
TCL% pred	nd	65.19 ± 14.62	—

Notes: nd, not determined.

Non smoker (NS), former smoker (FS), smoker (S).

Data are presented as mean ± SD. *p*-values were calculated by chi-square test or Student’s t-test.

## Discussion

IPF is a progressive aging-related disease characterized by the replicative senescence of lung epithelial cells, with lung epithelial II cell senescence being the key manifestation. Abnormally activated AECs induce the expansion and activation of fibroblast populations, causing deterioration of the lung structure (Jiang et al., 2017). Accumulating evidence indicates that senescent cells are metabolically active and secrete a large number of leukocyte mediators, including interleukin (IL)-1β, IL-6, and IL-8, which induce the differentiation of lung fibroblasts into myofibroblasts, thereby promoting IPF onset (Sugihara et al., 2020; Xu et al., 2020). Nevertheless, extensive validation is essential to better understand the role of cellular senescence in IPF pathogenesis.

Recent studies have explored the correlation between PBMCs, lung tissue RNAs, and IPF. For example, Herazo-Maya et al. (2013) performed a microarray expression profile analysis of RNAs expressed in PBMCs obtained from healthy individuals and patients with IPF, and identified some of the differentially expressed mRNAs that included a series of aging-related mRNAs, such as *CD28, ICOS, LCK,* and *ITK*. Moreover, Huang et al. (2014) revealed that the expression level of *LYCAT* was greatly reduced in the PBMCs and lung tissues of patients with IPF, as well as in a mouse model of bleomycin- and radiation-induced IPF. Hence, the PBMCs of patients with IPF were also used to verify the results of the bioinformatic analysis in this study. Gao et al. (2017) reported that the expression of the aging-related gene telomeric repeat-containing RNA (TERRA) in the PBMCs of patients with IPF was significantly enhanced and showed a substantial negative correlation with the percentage of predicted forced vital capacity. This finding might be attributed to the ability of *TERRA* to regulate the expression of telomeres and mitochondria, suggesting that aging plays an important role in IPF. Nevertheless, very few studies have explored this field, and further investigations are needed to better understand the role played by cellular senescence in IPF.

A few cancer-based studies have analyzed aging-related genes (Jia et al., 2018; Avelar et al., 2020; Song et al., 2020; Yue et al., 2021). For example, a recent study highlighted the role of 15 aging-related genes in colorectal cancer, among which some were associated with disease prognosis (Yue et al., 2021). However, a bioinformatic analysis of aging-related genes in IPF has not yet been performed. In the present study, 16 potential aging-related genes associated with IPF were identified using bioinformatic analysis. Of these, some genes have been studied previously. For instance, Hernandez et al. (2020) demonstrated that *IGF1* expression was upregulated in myofibroblasts and the fibrotic lung tissue by transforming growth factor β, whose expression was positively correlated with deteriorated lung function in advanced IPF; Guiot et al. (2016) found that the expression of *IGFBP2* was substantially higher in the serum of patients with IPF than that in healthy participants, but was reduced in the serum of patients receiving specific anti-fibrosis therapy. These results support the findings of our bioinformatic analysis and suggest that *IGFBP2* may be a promising biomarker of IPF. Moreover, Toren et al. (2021) investigated the relationship between lung fibrosis-related and longevity-associated genes in a mouse model of bleomycin-induced fibrosis; they highlighted age as an important risk factor for pulmonary fibrosis and found that pro-longevity genes tended to be related to anti-fibrosis. Our results are partially consistent with these findings. Notably, *KL* (Klotho) gene, which is a pro-longevity gene, was found to be overexpressed in healthy control lung tissues in our study. Consistently, (Toren et al., 2021) also demonstrated that *KL* is a pro-longevity and anti-fibrotic gene. However, other genes screened in our bioinformatic analysis were not mentioned in the study by Toren et al. This discrepancy could be due to the differences in the study design and aims of the two studies. We aim to explore more aging-associated genes that could potentially be related to IPF in the future.

The GO and KEGG enrichment analyses were also performed in this study to investigate the potential biological functions of these differentially expressed aging-related genes. Our results highlight the involvement of these genes in senescence and mitochondrial apoptosis. Consistently, accumulating evidence supports that IPF progression is affected by cellular senescence. For example, extracellular vesicles containing microRNA-23b-3p and microRNA-495-3p derived from lung fibroblasts increase reactive oxygen species levels in mitochondria and cause mitochondria-related damage in lung epithelial cells, resulting in DNA damage and subsequently, epithelial cell senescence (Kadota et al., 2020); another recent study reported that the PTEN/NF-κB pathway in senescent AECs facilitated the accumulation of collagen in fibroblasts, leading to lung fibrosis (Tian et al., 2019). Moreover, the KEGG pathway enrichment analysis suggested that these aging-related genes were mainly involved in endocrine resistance and the MAPK pathway. Endocrine resistance is a common diabetes-related phenomenon (Ahmad et al., 2014); diabetic lung damage is a part of the multi-system disease, and the lungs are one of the target organs of diabetic damage. Additionally, lung tissue abnormalities and pathophysiological changes caused by diabetes result in tissue fibrosis, such as diabetic nephropathy and diabetic cardiomyopathy. Notably, diabetes can greatly enhance the risk of pulmonary fibrosis, indicating that pulmonary fibrosis may be a complication of diabetes (Irfan et al., 2011; V. ; Kumar et al., 2020; Wang et al., 2020). Furthermore, Fang et al. (2018) demonstrated that p38 MAPK is essential for the epithelial–mesenchymal transition of AECs induced by Wnt1 and lithium chloride, and Antoniou et al. (2010) proposed the involvement of the MAPK signaling pathway in IPF pathogenesis. These findings are consistent with the results of our KEGG enrichment pathway analysis. However, further studies are needed to explore the biological functions of differentially expressed aging-related genes.

SVM-RFE is a machine learning technique based on an SVM, which searches for the best variable by subtracting the feature vector generated by the SVM (Wan et al., 2019). The advantage of the SVM is that an SVM classifier depends only on the support vectors, and the classifier function is not influenced by the entire dataset. LASSO regression is another machine learning technique that identifies the variable by finding the value of λ when the classification error is the least (Zhao et al., 2020). These two algorithms are primarily used to select characteristic variables and create the best classification model. Unlike the conventional univariate analysis, the LASSO regression used in this study aimed to select variables for logistic regression to avoid overfitting. After screening based on these two methods, six aging-related genes (*IGF1, RET, IGFBP2, CDKN2A, JUN,* and *TFAP2A*) were identified. The random forest algorithm provided an important metric for these aging-related hub genes. These genes could be used for improved IPF diagnosis; the ROC curves of GSE150910 and GSE32537 datasets had higher AUC values, suggesting that these six genes are potential diagnostic biomarkers for IPF.

To verify the results of the bioinformatic analysis, blood samples were collected from 20 patients with IPF and 20 healthy individuals, and qRT-PCR was performed to identify the six differentially expressed aging-related genes screened by LASSO regression and SVM-RFE. We observed that the expression levels of *IGF1, RET,* and *IGFBP2* in IPF blood samples were considerably enhanced compared to those in the blood samples of healthy individuals, while expression levels of *CDKN2A, JUN,* and *TFAP2A* were comparable between the two groups. Notably, studies have suggested that *IGF1, RET,* and *IGFBP2* are closely related to IPF. For example, Sun et al. (2021) reported that PI3K/AKT signal activation induced by *IGF1* was involved in the aging of type II AECs and IPF progression by inducing the release of connective tissue growth factor, transforming growth factor-beta 1, and matrix metalloproteinase 9; Tran et al. (2014) showed that continuous IGF1 treatment inhibited the biological activity of SIRT1 deacetylase, which induces p53 acetylation and increases the stability of p53 and biological activity, thereby enhancing senescence in immature cells; Guiot et al. (2017) reported that the concentration of insulin-like growth factor binding protein (IGFBP)-2 in the sputum supernatant of patients with IPF was greatly increased compared to that in healthy participants. The mechanism may be related to cell senescence with SASP, which involves the secretion of soluble factors, such as IGFBPs that perform extracellular and intracellular functions in an IGF-dependent or -independent manner. Interestingly, while extracellular IGFBP2 counter-regulates IGF-induced cell hyperproliferation, apoptosis is inhibited by intracellular IGFBP2 via its interaction with p21 to protect itself from ubiquitin-dependent degradation (Mercurio et al., 2020).

To date, there are no reports on the role of *RET* in IPF. *RET* is a new driver gene discovered after *EGFR* and *ALK* in non-small cell lung cancer. *RET* fusion may be responsible for the pathogenesis in patients with lung cancer without mutations in *EGFR, ALK,* or *ROS1*, which may be related to the *RET-*induced promotion of apoptosis resistance (Drilon et al., 2020). Additionally, the experimental verification results of *JUN* and *TFAP2A* differed from the results of biological information analysis, probably due to the small sample size in this study. Moreover, no major difference was observed in *CDKN2A* mRNA expression between patients with IPF and healthy participants in this study. However, Du et al. (2018) showed that the *CDKN2A* expression level was lower in the peripheral blood of patients with IPF, whereas it was higher in healthy controls. This finding is different from the results of our present study; this discrepancy could be due to the differences in the sample source and the composition of study participants. The study by Du et al. included only male patients, whereas both male and female patients were included in our study. In addition, IPF severity differed in patients of both the studies. Furthermore, this study had a smaller sample size of only 20 cases and different experimental operators and varied experimental conditions in the two studies might have caused a variation in the results. Hence, studies with larger sample sizes should be designed in the future to further confirm the findings of our study.

This study has some limitations. First, the results of bioinformatic analysis were based on data obtained from IPF and normal lung tissues; however, due to technical reasons, we could not obtain the lung tissue of patients with IPF in our hospital, and the results could only be verified experimentally through blood samples. Moreover, we were unable to analyze differential gene expression between old and young patients with IPF because most of the patients included in this study were middle-aged or old. In the future, we intend to study the differentially expressed genes between old and young patients with IPF. Second, the sample size was small because fewer clinical samples were included in this study. Hence, our conclusions need to be confirmed using a larger IPF cohort. Third, only the expression levels of differentially expressed aging-related genes were verified in the clinical samples, and the mechanisms underlying the functions of these genes were not explored in IPF cells or mouse models. Thus, a more detailed investigation is required in the future.

In conclusion, six potential aging-related genes associated with IPF were identified in this study using bioinformatic analysis and machine learning methods. The prognostic role of key genes *IGF1, RET,* and *IGFBP2* was verified using clinical samples. These genes may affect the occurrence and prognosis of IPF by regulating senescence. These present findings improve our knowledge regarding IPF and may help design treatment strategies for this disease in the future.

## Data Availability

The original contributions presented in the study are included in the article/Supplementary Material, further inquiries can be directed to the corresponding author.
