# Supplementary figures and images for: Identification and Validation of Aging-Related Genes in Idiopathic Pulmonary Fibrosis
Source: Front Genet. 2022 Feb 8;13:780010. doi: 10.3389/fgene.2022.780010 (PMC8863089; doi:10.3389/fgene.2022.780010)

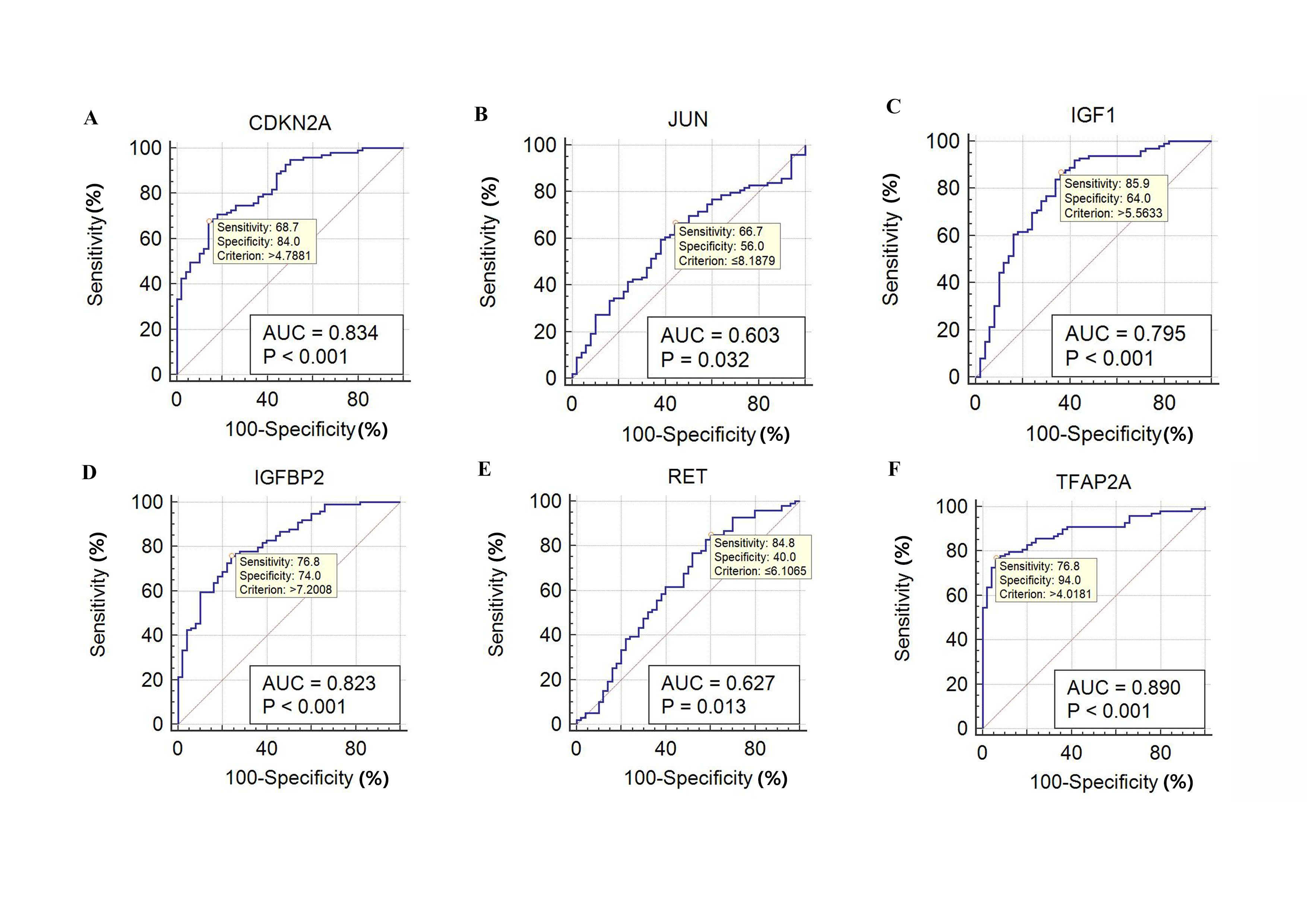

Supplement: Supplementary file 1 [file Image3.TIF]

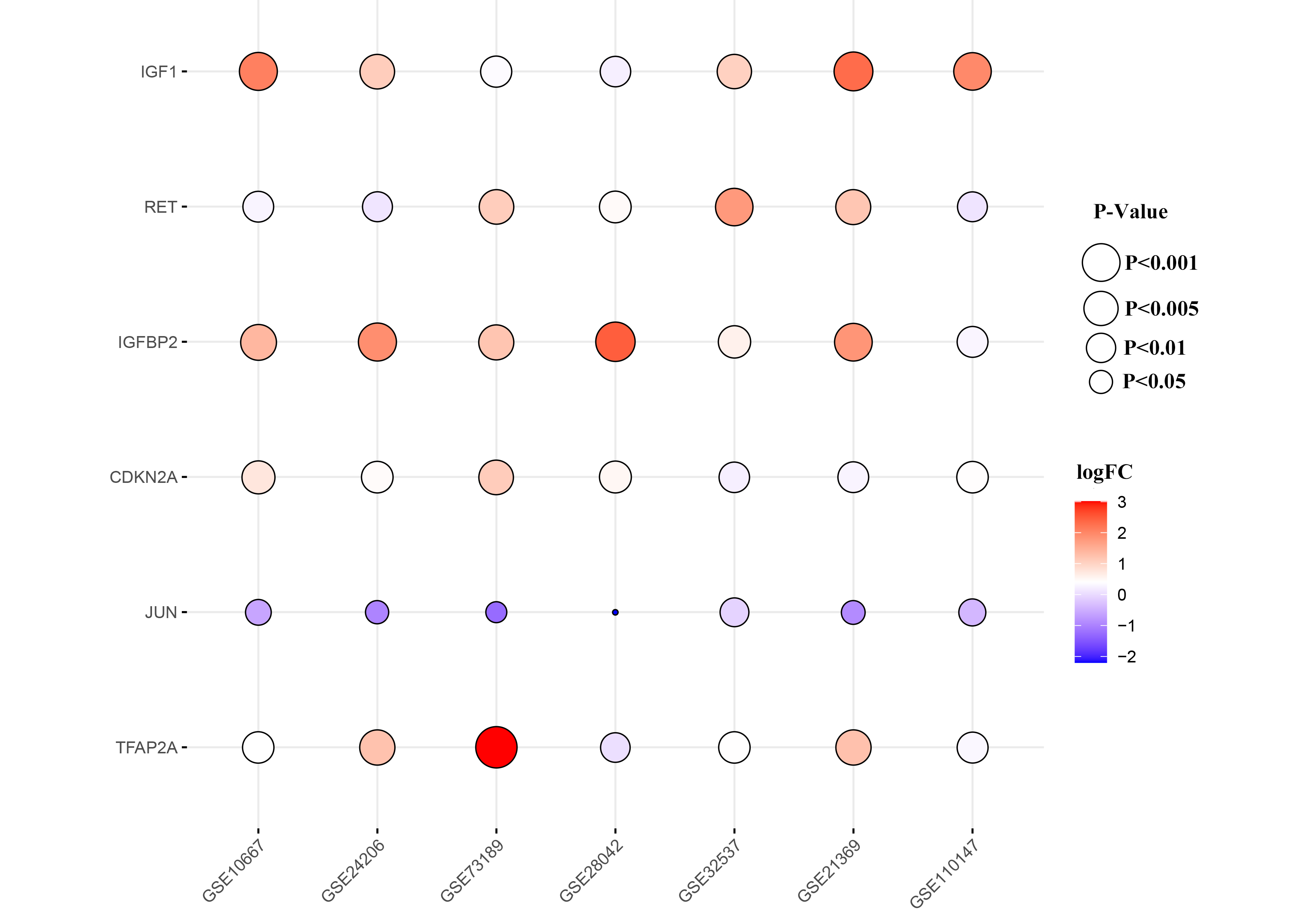

Supplement: Supplementary file 2 [file Image2.TIF]

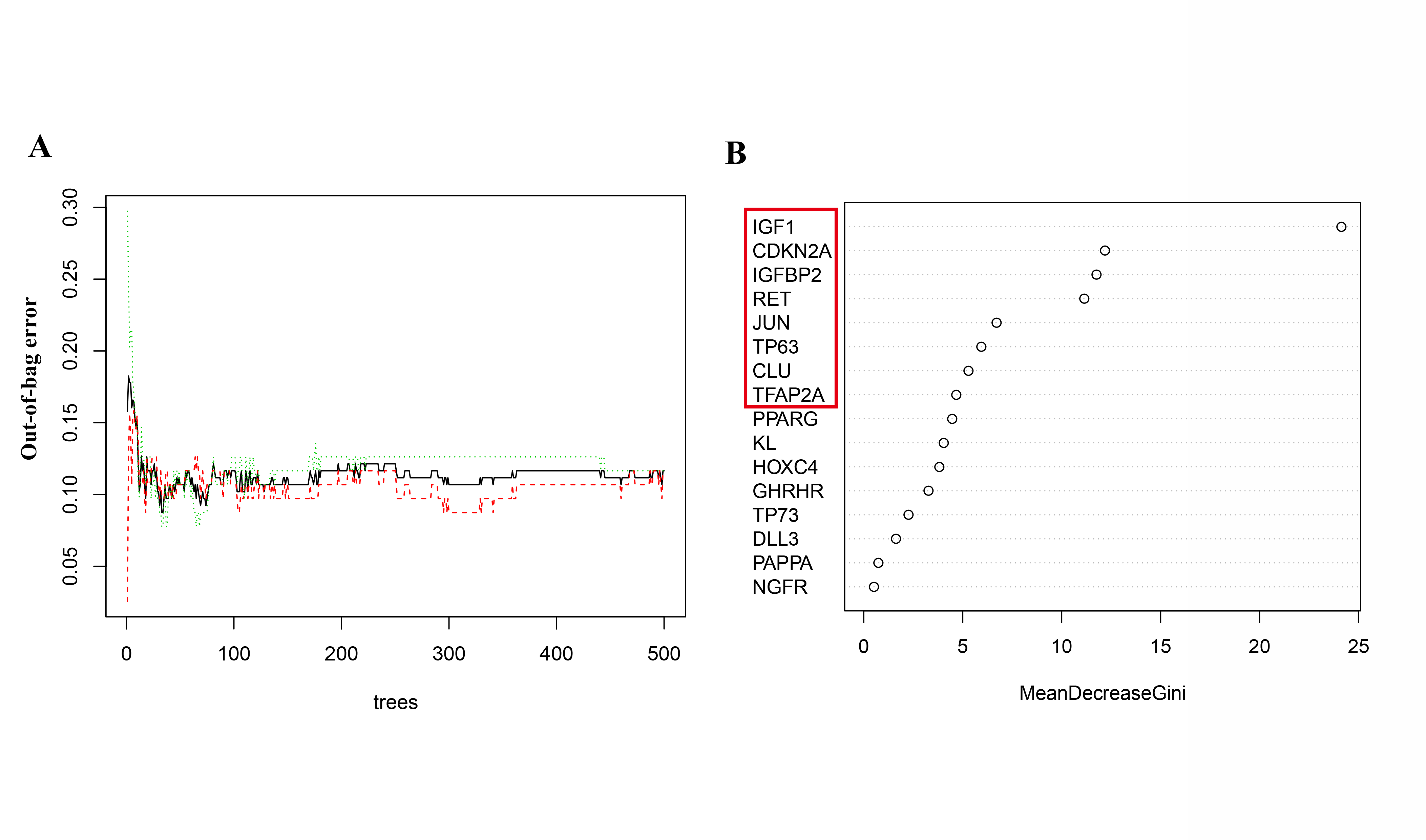

Supplement: Supplementary file 3 [file Image1.TIF]
