# Supplementary material for: Identification and Validation of Aging-Related Genes in Idiopathic Pulmonary Fibrosis
Source: Front Genet. 2022 Feb 8;13:780010. doi: 10.3389/fgene.2022.780010 (PMC8863089; doi:10.3389/fgene.2022.780010)
Supplement: Supplementary file 4 [file DataSheet1.docx]

Supplementary File 1

###For differentially expressed genes analysis formula and R code

library("limma")

library(pheatmap)

setwd("C:\\Users\\DELL\\Desktop\\GSE150910 ")

inputFile="SFexp.txt" #

fdrFilter=0.05 #fdr

logFCfilter=1 #logFC

conNum=103

treatNum=103

# Read the file

outTab=data.frame()

grade=c(rep(1,conNum),rep(2,treatNum))

rt=read.table(inputFile,sep="\t",header=T,check.names=F)

rt=as.matrix(rt)

rownames(rt)=rt[,1]

exp=rt[,2:ncol(rt)]

dimnames=list(rownames(exp),colnames(exp))

data=matrix(as.numeric(as.matrix(exp)),nrow=nrow(exp),dimnames=dimnames)

data=avereps(data)

data=data[rowMeans(data)>0,]

data[data<0]=0

# Differential gene analysis

for(i in row.names(data)){

geneName=unlist(strsplit(i,"\\|",))[1]

geneName=gsub("\\/", "_", geneName)

rt=rbind(expression=data[i,],grade=grade)

rt=as.matrix(t(rt))

wilcoxTest<-wilcox.test(expression ~ grade, data=rt)

conGeneMeans=mean(data[i,1:conNum])

treatGeneMeans=mean(data[i,(conNum+1):ncol(data)])

logFC=log2(treatGeneMeans)-log2(conGeneMeans)

pvalue=wilcoxTest$p.value

conMed=median(data[i,1:conNum])

treatMed=median(data[i,(conNum+1):ncol(data)])

diffMed=treatMed-conMed

if( ((logFC>0) & (diffMed>0)) | ((logFC<0) & (diffMed<0)) ){

outTab=rbind(outTab,cbind(gene=i,conMean=conGeneMeans,treatMean=treatGeneMeans,logFC=logFC,pValue=pvalue))

}

}

pValue=outTab[,"pValue"]

fdr=p.adjust(as.numeric(as.vector(pValue)),method="fdr")

outTab=cbind(outTab,fdr=fdr)

#result

write.table(outTab,file="all.xls",sep="\t",row.names=F,quote=F)

# result

outDiff=outTab[( abs(as.numeric(as.vector(outTab$logFC)))>logFCfilter & as.numeric(as.vector(outTab$fdr))<fdrFilter),]

write.table(outDiff,file="diff.xls",sep="\t",row.names=F,quote=F)
